# The type-VI secretion system of the beneficial symbiont *Vibrio fischeri*

**DOI:** 10.1099/mic.0.001302

**Published:** 2023-02-21

**Authors:** Kirsten R. Guckes, Tim I. Miyashiro

**Affiliations:** ^1^​ The Microbiome Center, Huck Institutes of the Life Sciences, Pennsylvania State University, PA, USA

**Keywords:** *Euprymna scolopes*, microbial competition, symbiosis, type VI secretion system, *Vibrio fischeri*

## Abstract

The mutualistic symbiosis between the Hawaiian bobtail squid *Euprymna scolopes* and the marine bacterium *Vibrio fischeri* is a powerful experimental system for determining how intercellular interactions impact animal–bacterial associations. In nature, this symbiosis features multiple strains of *V. fischeri* within each adult animal, which indicates that different strains initially colonize each squid. Various studies have demonstrated that certain strains of *V. fischeri* possess a type-VI secretion system (T6SS), which can inhibit other strains from establishing symbiosis within the same host habitat. The T6SS is a bacterial melee weapon that enables a cell to kill adjacent cells by translocating toxic effectors via a lancet-like apparatus. This review describes the progress that has been made in understanding the factors that govern the structure and expression of the T6SS in *V. fischeri* and its effect on the symbiosis.

## The type-VI secretion system as a weapon for bacterial symbionts

The type-VI secretion system (T6SS) is a molecular lancet that enables bacteria to engage in competitive interactions by delivering toxins into adjacent cells [1]. The T6SS is encoded by many Gram-negative bacteria and translocates effector proteins from the cytoplasm into the extracellular environment. If a cell possessing a T6SS is in contact with another cell, then the translocation event can deliver effectors into the neighbouring cell. Because many effectors are potent toxins that target and disrupt the functions of specific cellular structures, T6SS-mediated delivery of those effectors into another cell often leads to cellular damage that is lethal. Consequently, the T6SS is a powerful mechanism that facilitates contact-dependent competition within polymicrobial environments. This review focuses on the T6SS of the bacterial symbiont *V. fischeri* that promotes intraspecific competition between strains.

### Competitive interactions in microbial ecosystems

The surface of host tissue provides a natural arena for bacterial cells to interact with one another [2–5]. Such intercellular interactions can facilitate ecological cooperation and competition, which in turn can influence the local composition of bacterial populations and consortia [6, 7]. While cooperative interactions between various taxa have the potential to increase metabolic efficiency within an ecological network, they tend to introduce strong positive feedbacks that threaten network stability [8]. By dampening the strength of such positive feedback, competitive interactions are thought to play a particularly important role in stabilizing the networks of microbial ecosystems, including the complex microbiomes of animals [7, 8].

The resources that dictate bacterial fitness within an ecosystem are nutrients, energy sources and space. In the constant struggle for these resources, two modes of competition have arisen among bacteria: exploitation and interference [6]. Exploitative competition occurs when one bacterium indirectly affects the fitness of the another through resource management, e.g. by scavenging a micronutrient that is essential for the growth of the other bacterium [9]. In contrast, interference competition describes interactions whereby bacteria directly inflict damage to their competitors [10]. The specific molecular mechanisms underlying interference competition are multifaceted and vary in both striking distance and specificity. Some interference competition mechanisms function locally through direct contact, which is the case for contact-dependent growth inhibition systems (CDIs), which deliver surface-exposed toxins to competitor cells [11]. In contrast, other mechanisms are diffusible, enabling them to be effective against competitor cells at a distance, such as secreted antimicrobial peptides [12]. Competition mechanisms can also act inter- or intra-specifically, which affects the overall diversity of bacteria that coexist within an ecosystem. Certain mechanisms enable competition for a particular niche by impacting related bacterial cells, e.g. bacteriocins and type-V secretion system CDIs [11, 13]. The T6SS is a contact-dependent mechanism that can introduce interference competition among a wide-range of cell types; however, here, the focus is largely on intra-specific competition.

### Structure and function of the type-VI secretion system

**Fig. 1.**
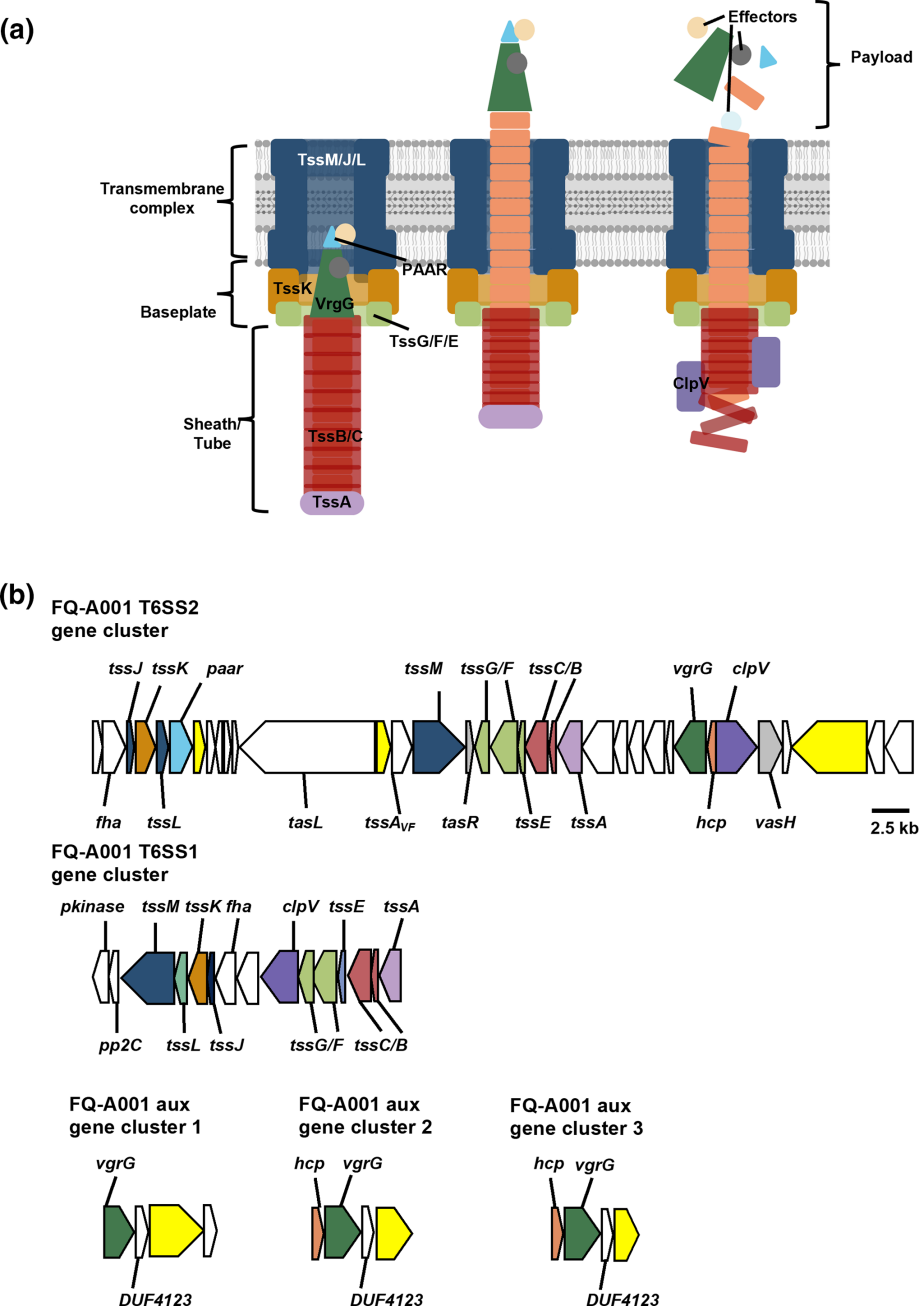
Structure and function of the type-VI secretion system (a) Illustration of the T6SS structure in multiple phases of assembly and effector delivery. Components assemble with the sheath in the uncontracted state (left). As the sheath contracts, the needle traverses both membranes (middle). Outside of the cell, the tip of the needle disassembles to deliver the effector payload, which includes structural proteins Hcp and VgrG. The other components are recycled via the ClpV ATPase (right). This figure was created using Biorender.com. (b) The T6SS gene clusters from FQ-A001 with all core components labelled. Colours correspond to the illustration in (a), except for regulators which are shown in grey. White genes are genes contained within the gene cluster but are not shown in the illustration present in (a). Yellow genes represent putative effectors.

Understanding of the structure-function relationships of T6SSs has emerged from a variety of bacterial species, including *Pseudomonas aeruginosa* [14–16]*, Escherichia coli* [17–19]*, Vibrio cholerae* [20–22] and *Burkholderia* [23–26]. Once fully assembled at the cell wall, a T6SS consists of multiple copies of 13 distinct subunits that form three primary complexes: a transmembrane complex, a baseplate and a sheath/tube complex (Fig. 1a). The 13 core components are labelled with the suffix Tss, which stands for Type six secretion (Table 1) [1], and their genes are typically co-localized within gene clusters (e.g. Fig. 1b) [27, 28]. Approximately 25 % of all sequenced Gram-negative bacteria encode these gene clusters [27], which suggests that T6SSs function in a plethora of ecological networks.

**Table 1.** Gene content for T6SS gene clusters in T6SS2-positive *V. fischeri* strain FQ-A001

**Core component**	**Vas homolog**	**Alternative/** **common name**	**T6SS1 gene locus**	**T6SS2 gene locus**	**Aux cluster 1**	**Aux cluster 2**	**Aux cluster 3**	**Predicated function**
TssA	VasJ	BimE/SciA	VFFQA001_05500	VFFQA001_15570				Aids in assembly [38]
TssB		VipA	VFFQA001_05495	VFFQA001_15565				Composes sheath [107]
TssC		VipB	VFFQA001_05490	VFFQA001_15560				Composes sheath [107]
TssD		Hcp		VFFQA001_15605		VFFQA001_078340	VFFQA001_16800	Composes inner tube [108]
TssE	VasS	HsiF	VFFQA001_05485	VFFQA001_15555				Composes baseplate sub-structure [109]
TssF	VasA		VFFQA001_05480	VFFQA001_15550				Composes baseplate sub-structure [110]
TssG	VasB		VFFQA001_05475	VFFQA001_15545				Composes baseplate sub-structure [110]
TssH	VasG	ClpV	VFFQA001_05470	VFFQA001_15610				AAA +ATPase [40]
TssI		VgrG		VFFQA001_15600	VFFQA001_06830	VFFQA001_07835	VFFQA001_16795	Composes spiked tip [111]
TssJ	VasD	SciN	VFFQA001_05450	VFFQA001_15475				Composes transmembrane sub-structure [29]
TssK	VasE		VFFQA001_05485	VFFQA001_15480				Composes baseplate sub-structure [110]
TssL	VasF	IcmH	VFFQA001_05480	VFFQA001_15485				Composes transmembrane sub-structure [29]
TssM	VasK	IcmF	VFFQA001_05435	VFFQA001_15535				Composes transmembrane sub-structure [29]
**Accessory/** **Other Component**	**Vas homolog**	**Alternative/** **Common name**	**T6SS1 gene locus**	**T6SS2 gene locus**	**Aux cluster 1**	**Aux cluster 2**	**Aux cluster 3**	**Predicated Function**
PAAR				VFFQA001_15490				Sharpens spiked tip [112]
DUF4123		Tap-1			VFFQA001_06835	VFFQA001_07830	VFFQA001_16790	Loads effector onto spike [113]
VasH				VFFQA001_15615				Promotes transcription of *hcp* [72]
TasR				VFFQA001_15540				Promotes transcription of *hcp* [97]
TasL				VFFQA001_15520				Promotes cell-cell contact [114]
Fha			VFFQA001_05455	VFFQA001_15470				Regulates T6SS assembly in a Thr phosphorylation pathway [115]
Pkinase			VFFQA001_05425					Regulates T6SS assembly as a kinase in a Thr phosphorylation pathway [116]
Pp2C			VFFQA001_05430					Regulates T6SS assembly as a phosphatase in a Thr phosphorylation pathway [116]
TssA_VF_				VFFQA001_15530				Aids in assembly [38]

The transmembrane complex defines the location of the T6SS within the cell wall and spans both inner and outer membranes (Fig. 1a). TssM, TssJ and TssL make up the transmembrane complex and assemble with fivefold symmetry to form a chamber that anchors the T6SS to the cell wall [29–31]. The sheath/tube complex and parts of the baseplate resemble and are evolutionarily related to components of a contractile phage tail [32]. The inner tube portion of the sheath/tube complex is composed of hexameric rings of TssD, which is commonly referred to as haemolysin coregulated protein (Hcp) [14]. TssB/C assemble with complementary sixfold symmetry around the Hcp tube to form the outer sheath [33]. The tube is capped by a spike that is formed by a trimer of TssI, which is commonly called Valine-glycine repeat G (VgrG) protein [34–36], and the tip of the spike is sharpened by another protein containing a proline-alanine-alanine-arginine (PAAR) motif that folds into a cone [37]. The tube/sheath complex polymerizes at the baseplate complex, which is composed of the TssE, TssF, TssG and TssK [38]. TssA aids in assembly by initiating recruitment of the baseplate and coordinating elongation of the tube/sheath complex [39]. The baseplate anchors the sheath/tube complex to the transmembrane complex at the cytoplasmic face of the inner membrane, with the spiked tip of the inner tube pointing away from the cell. Contraction of the sheath thrusts the inner tube first through the central channel formed by the baseplate, then through the chamber formed by the transmembrane complex, and finally into the extracellular environment [40]. The components of the apparatus are recycled by the AAA +ATPase TssH, also referred to as ClpV [41].

Effector proteins are loaded onto the inner tube during the assembly of the T6SS, which enables their translocation into the extracellular space following contraction of the sheath. There are two types of effectors: specialized (or evolved) and cargo [42, 43]. A specialized effector is a VrgG, Hcp or PAAR protein that contains an additional domain with enzymatic activity [44, 45]. Consequently, specialized effectors contribute to both the structural integrity of a T6SS and its killing activity towards competitors. In contrast, cargo effectors are noncovalently attached to Hcp, VgrG or a PAAR-containing protein that has already been incorporated into T6SS [42, 46–48]. Loading of certain cargo effectors requires specific adaptor proteins or chaperones, which disassociate from the T6SS prior to the translocation event and are thus not part of the effector payload [42]. Following translocation, the effectors and remaining components of the inner tube disassociate into the extracellular environment. In the absence of a neighbouring cell, some effectors can promote contact-independent toxicity or metal acquisition by acting in the extracellular milieu [49, 50]. However, if the translocation event involves a neighbouring cell, then the effectors can act upon intracellular targets. While some effectors cause toxicity in eukaryotic cells [51–53], most effectors exhibit antimicrobial activity [42], with specific targets including components of the cytoplasmic membrane [54], peptidoglycan [55] or nucleic acids [56, 57]. Self-intoxication and killing of kin by T6SS-positive cells are prevented by the expression of a cognate immunity factor for each effector that specifically neutralizes its activity. Thus, the susceptibility of a cell to T6SS-dependent killing is dictated by the ability to express immunity factors corresponding to the specific effectors being translocated [42].

### *Vibrio fischeri* as a model symbiont for T6SS-dependent interactions

The partnership between the Hawaiian bobtail squid, *Euprymna scolopes*, and the bacterial symbiont *V. fischeri* (a.k.a. *Aliivibrio fischeri *) has recently emerged as an experimental platform for studying the intercellular interactions that take place within a host [58, 59]. *E. scolopes* houses multiple populations of *V. fischeri* within a specialized symbiotic organ referred to as the light organ that is located within the mantle cavity. *V. fischeri* is a bioluminescent bacterium, and the bioluminescence produced while in symbiosis with *E. scolopes* enables the nocturnal squid to eliminate its shadow within the water column and camouflage itself from predators [60]. Bioluminescence is a byproduct of the enzyme luciferase, which is encoded within an operon called the *lux* locus. Transcriptional activity of the *lux* locus depends on quorum sensing, which describes the signalling mechanism based on diffusible molecules called autoinducers [61]. Conditions that promote a high concentration of autoinducer, e.g. high cell densities, will trigger signalling pathways that promote bioluminescence production [61]. Each dawn, the squid expels approximately 90 % of established light organ symbionts, and the cells grow to high cell densities that promote quorum sensing and bioluminescence production [62, 63]. This expulsion behaviour destabilizes the spatial structure of light organ populations, which can affect the subsequent dynamics of the symbiont populations, including intercellular interactions.

**Fig. 2.**
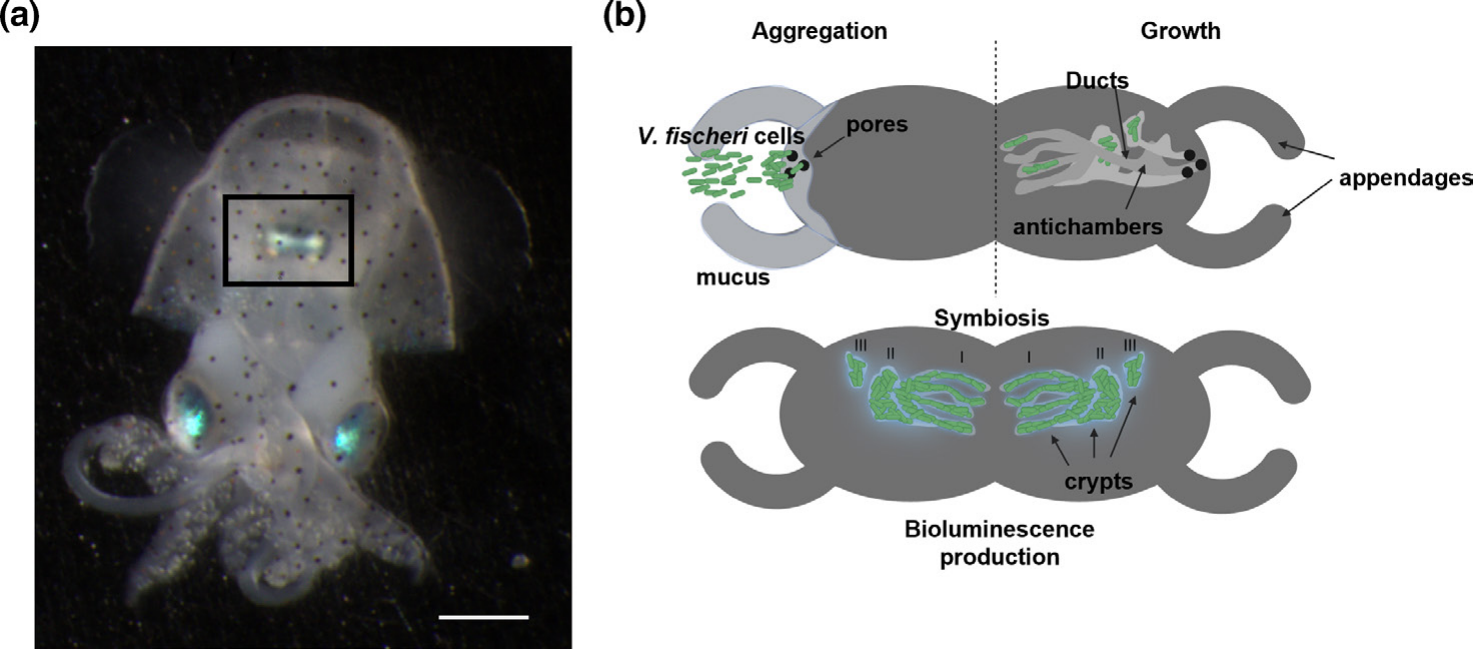
Symbiosis establishment between *Euprymna scolopes* and *V. fischeri* (a) Image of a juvenile animal with the light organ highlighted by the black box. Scale bar represents 1 mm. (b) Illustration of the processes leading to symbiosis establishment. Top left image shows bacterial cells collecting near the mucus-covered nascent light organ. Cells aggregate near pores located between the appendages. Cells enter the pores and traverse ducts that lead to individual crypts. In total, one–two cells enter each crypt. Once inside the crypts, cells use host-derived nutrients to replicate. Bottom image shows the result of cellular replication. Bacterial cells fill each the three crypts on each side of the light organ and begin to produce bioluminescence as cell number and quorum-sensing signalling molecules increase. This figure was created using Biorender.com.

**Fig. 3.**
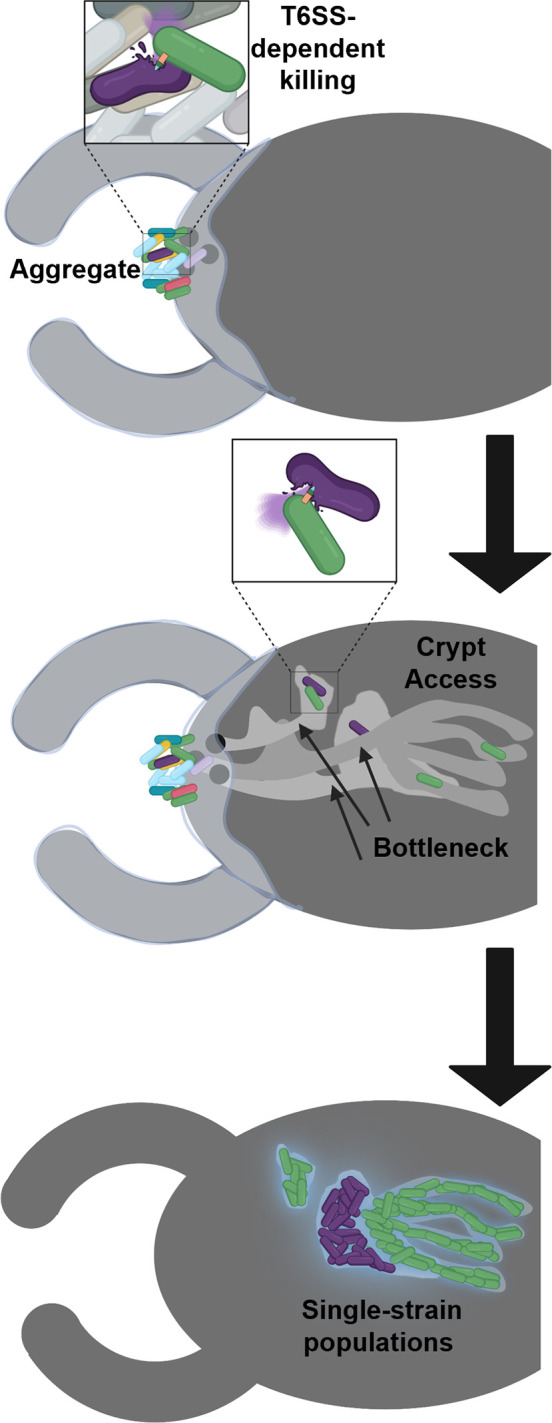
Model of T6SS impact on symbiotic *V. fischeri* populations Illustration of cellular interactions during symbiosis establishment. The left half of the bilaterally symmetrical light organ is being shown. Black arrows represent the passage of time to show multiple steps of the colonization process. Top image shows T6SS-dependent killing in aggregate formed outside of light organ pores. Cellular interactions occur within mucus on the light organ exterior. Middle image shows the interactions that occur within light organ crypts. Purple cells are susceptible to the T6SS of green cells, which cause their elimination. The bottom image shows the point at which the bacteria have established symbiosis as depicted by the production of light. In these crypts, populations are all composed of a single strain. This figure was created using Biorender.com.

The symbiosis is initiated in juvenile animals (Fig. 2a), with each hatchling acquiring environmental *V. fischeri* cells to establish symbiotic populations that are maintained over the lifetime of the host [64, 65]. Adult wild-caught animals harbour five–six genetically distinct strains [66], which suggests that multiple strains initially colonize the light organ. The nascent light organ exhibits bilateral symmetry, with each side featuring three epithelium-lined crypt spaces that serve as independent colonization sites. Colonization of the light organ can be described according to discrete stages, which have been the subject of multiple reviews [58, 59]. There are three stages when significant intercellular interactions between bacterial cells occur: aggregation, growth and symbiosis (Fig. 2b). During the aggregation stage, environmental bacteria directly interact with one another while forming large cellular aggregates on the surface of the light organ (Fig. 2b) [67]. Over time, these aggregates become enriched with *V. fischeri* cells that become primed for entering the light organ [68]. Cells access the crypt spaces via a migration path through ducts that connect external pores to the light organ interior [69]. As the cells enter the colonization site, this pathway is constricted by the host, which forms a host-imposed bottleneck (Fig. 3) [70]. During the growth stage, the cells that have accessed crypt spaces grow on host-derived nutrients [71–73], which leads to rapid clonal expansion until the corresponding crypt spaces are filled (Fig. 2b). Consequently, the resulting populations feature cells at high density and in direct contact with one another. Furthermore, crypt spaces that are initially accessed by more than one strain will subsequently feature mixed populations that permit direct intercellular interactions between different strain types (Fig. 3) [66]. Finally, during the symbiotic stage, the *V. fischeri* populations are at high cell density within the crypt spaces and produce bioluminescence in response to quorum sensing (Fig. 2b). Recently, this form of intercellular interaction was also shown to occur between the populations of different crypt spaces, with each population able to detect autoinducer produced by populations in other crypt spaces [74]. The colonization bottleneck prevents other *V. fischeri* cells from colonizing an animal that has already established symbiosis [70, 75], which highlights the critical nature of the interactions that take place during the initial stages of colonization. Thus, the initial steps of light organ colonization provide opportunities to investigate the molecular mechanisms by which intercellular interactions contribute to symbiosis establishment.

Evidence of interference competition within the light organ first arose from squid colonization assays that used inoculums comprising strains FQ-A001 and ES114 [76]. While animals could be colonized by both strains, their crypt spaces were monospecific, i.e. they contained either FQ-A001 or ES114 but not both, and this phenomenon was termed strain incompatibility [76]. In subsequent studies, colonization assays using various mutants of FQ-A001 that are impaired for T6SS activity resulted in crypt spaces co-colonized with both strains, which demonstrated that the strain incompatibility depends on the T6SS of FQ-A001 [77–79]. Together, these studies pioneered the T6SS as a new area of research associated with the *E. scolopes–V. fischeri* symbiosis.

## Genetic organization of the T6SS in *V. fischeri*

Whole-genome sequencing revealed that a subset of *V. fischeri* strains, including FQ-A001, encode multiple T6SS gene clusters [77, 80]. The majority of genes that encode the T6SS of FQ-A001 are found within a single genetic locus that is referred to as T6SS2 due to its position on chromosome II (Fig. 1b) [79]. This gene cluster is encoded by approximately 75 % of the 81 *V *. *fischeri* genomes that have been published [81]. The T6SS2 gene cluster encodes for the 13 core components, as well as six accessory factors: lipoprotein TasL, transcription factors TasR and VasH, forkhead-associated domain protein (Fha), PAAR-containing protein, Pkinase and Pp2C phosphatase (Fig. 1 and Table 1) [82]. ES114, which can be killed by FQ-A001 in a T6SS2-dependent manner [79], is an example of a T6SS2-negative strain, i.e. one that does not have the T6SS2 gene cluster. In addition, ES114 does not exhibit killing activity towards other strains of *V. fischeri *, which has led to its frequent use as a competitor in culture-based assays that test for T6SS activity in other strains [77–79, 83–86]. In fact, these assays readily identify strains that encode the T6SS2 gene cluster through their inhibition of ES114 growth [79, 82], which provides further evidence that T6SS2 represents a mechanism of interference competition that is widespread among symbiotic strains of *V. fischeri *.

All sequenced *V. fischeri* genomes, including those of T6SS2-negative strains like ES114, feature a cluster of genes on chromosome I that are annotated with functions related to T6SS activity, which led to its designation as the T6SS1 gene cluster [79]. While the T6SS1 gene cluster encodes 11 core components, it does not contain *hcp* or *vgrG* genes, which is noteworthy because both factors are necessary for a functional T6SS. One of the genes within the T6SS1 gene cluster was disrupted with no effect on T6SS activity [79]. Thus, one possibility is that the T6SS1 gene cluster does not contribute to T6SS activity; however, this conclusion is premature for several reasons. First, genomes of T6SS-positive bacteria frequently feature auxiliary gene clusters that encode Hcp and VgrG in addition to specific effectors. For instance, the genome of FQ-A001 features three auxiliary T6SS gene clusters with different VgrG homologs (Fig. 1b). Two of these gene clusters also encode an Hcp homolog. Therefore, T6SS2-negative strains may depend on auxiliary gene clusters for the core components that are missing from the T6SS1 gene cluster. Second, a report involving transcriptomic studies of ES114 shows low transcriptional levels of the T6SS1 gene cluster [87], which suggests that the environmental conditions that can activate these genes in culture have yet to be identified, which would prevent the detection of T6SS activity associated with this genetic locus. Finally, it is possible that a subset of the genes within the T6SS1 gene cluster provide functional redundancy or alternative functions for the T6SS encoded by the T6SS2-positive strains. Therefore, further work is warranted to determine how the T6SS1 gene cluster affects the cellular physiology of *V. fischeri* and whether it plays any role in intercellular interactions.

## Activity and function of the T6SS of *V. fischeri*

Culture-based studies have shown that T6SS2-positive strains FQ-A001 and ES401 each inhibit the growth of ES114 *in vitro* [77–79]. Genetic mutants of ES401 and FQ-A001 strains (as described below) have been used to demonstrate that the inhibition of ES114 depends on the T6SS [77–79].

### Activity and function of T6SS core components

Historically, the first T6SS gene to be targeted for disruption in *V. fischeri* was *tssF* (*vasA*) [79]. *V. fischeri* contains two genes that encode TssF, with one in the T6SS2 gene cluster (*VFFQA001_05480*) and the other in the T6SS1 gene cluster (*VFFQA001_15550*) (Table 1 and Fig. 1b). The TssF baseplate protein contains three domains: (1) an N-terminal α-helix; (2) a central domain composed of three β-barrels; and (3) a C-terminal domain that, along with TssG, forms a triangular core structure [88]. Both VFFQA001_05480 and VFFQA001_15550 are predicted to contain these three domains and fold into a structure resembling the phage baseplate wedge protein gp6 [88, 89], which are consistent with putative structural roles for the baseplate (Fig. 1a) [90]. A knockout mutant for *tssF* within the T6SS2 gene cluster fails both to assemble sheaths and to inhibit the growth of ES114 *in vitro* [79], which suggests the TssF homologue of T6SS2 contributes to T6SS activity in FQ-A001. Furthermore, this mutant could co-colonize crypt spaces with ES114, which revealed a role of T6SS2 in mediating interactions between FQ-A001 and ES114 *in vivo* [79]. In contrast, a mutant with the T6SS1 *tssF* gene knocked out had no effect on ES114 growth [79], which suggests that this TssF homologue does not contribute to the T6SS activity observed in FQ-A001.

Another T6SS component that has been used to define T6SS activity in *V. fischeri* is Hcp [77], which is the primary subunit of the inner tube that translocates effectors across the cell wall [91]. The genome of the T6SS2-positive strain FQ-A001 has two genes encoding identical copies of Hcp. *VFFQA001_15605* (*hcp*) is located within the T6SS2 gene cluster, and *VFFQA001_07840* (*hcp1*) is located within auxiliary cluster 2 on Chromosome I (Fig. 1b). The *hcp* and *hcp1* genes are functionally redundant, and the deletion of both genes was necessary to generate a mutant that cannot kill ES114 *in vitro* or exhibit strain incompatibility *in vivo* [77]. Hcp monomers are composed of two β-sheets formed by four or five β-strands folded onto each other to form a β-barrel fold that is stabilized by the hydrophobic residues at its core [18]. The Hcp protein encoded by *hcp* and *hcp1* is predicted to fold similarly to the Hcp homologue of *V. cholerae* [89, 92], which supports a similar role in *V. fischeri* for forming the inner tube. FQ-A001 encodes a third *hcp* gene (*VFFQA001_16800*) within auxiliary cluster 3 that is predicted to encode another Hcp homologue that has 58% identity to those encoded by *hcp*/*hcp1*. Notably, the observation that an *hcp*/*hcp1* knockout mutant (Hcp^-^) does not kill ES114 [77] suggests that the third allele does not contribute significantly to T6SS activity in *V. fischeri *. However, further experimentation involving mutagenesis of *VFFQA001_16800* is necessary to test this hypothesis directly.

The *VFFQA001_15535* gene encodes TssM, which is a core component of the TssJLM membrane complex that promotes initial assembly of the T6SS, anchors the baseplate to the cell wall, and directs the inner tube during translocation (Table 1 and Fig. 1a). Consistent with other TssM homologues, the 1132-residue VFFQA001_15535 encodes three transmembrane domains (residues 15–33, 38–58 and 402–422), with the C-terminal end of the protein predicted to localize to the periplasmic space. Despite low amino acid identity to the TssM homologue of enteroaggregative *E. coli *, the periplasmic domain of VFFQA001_15535 is predicted to fold similarly, which would permit interaction with the outer membrane lipoprotein TssJ to form a channel for the inner tube to pass through during a translocation event [29]. Residue 815 of the TssM of *V. fischeri* corresponds to a conserved asparagine that is predicted to form part of a periplasmic gate with a pore too small for inner tube components to pass during its assembly [93]. Consistent with this integral role of TssM in T6SS assembly, a mutant of ES401 containing a Tn insertion within *tssM* failed to form sheaths and could not inhibit ES114 growth [94]. The cytoplasmic portion of TssM (residues 61–401) also features Walker A and Walker B motifs, which are highly conserved GTP-binding regions in GTPase family members. These nucleotide-binding motifs are found in some but not all TssM homologues. In *Agrobacterium tumefaciens *, both motifs enable the corresponding TssM to bind and hydrolyse ATP, which recruits Hcp to the TssM-TssL base of the membrane complex within the inner membrane [95]. Furthermore, Hcp secretion by *A. tumefaciens* depends on TssM exhibiting full ATPase activity [95]. In contrast, substitution of the residues within the Walker A motif of EvpO, which is the TssM homologue in *Edwardsiella tarda *, did not affect the profile of extracellular proteins secreted by its T6SS [96]. Consequently, future biochemical and genetic experiments that target the nucleotide-binding motifs of TssM are necessary to determine whether they play a role in T6SS assembly and function.

### Activity and function of T6SS accessory components

The T6SS2 gene cluster of FQ-A001 contains two genes that each encode an N-terminal ImpA_N domain: *VFFQA001_15570* and *VFFQA001_15530* (Table 1). In addition to the ImpA_N domain, VFFQA001_15570 also features a middle domain (Nt2) and C-terminal domain (CTD) that classify this protein as a TssA2-type homologue [39]. TssA2 proteins interact with components of the baseplate, inner tube, sheath and cytoplasmic components of the transmembrane complex to prime the initial assembly and polymerization of the inner tube and sheath at the baseplate. A conserved WEP motif within the CTD of VFFQA001_15570 (residues 479–481) suggests that this domain forms dimers that further assemble a ring with either five-point (decamer) or six-point (dodecamer) symmetry to cap the cytoplasmic end of the inner tube/sheath complex [39]. The presence of a 25-residue C-terminal extension downstream of the CTD supports the six-point symmetry, although experimentation is necessary to test that model.

The other ImpA_N-domain protein VFFQA001_15530 features a middle 23-residue hydrophobic domain that classifies this protein as a homologue of TagA, which localizes directly to the cytoplasmic membrane to control the extent of polymerization [97]. More specifically, when polymerization extends the inner tube/sheath complex to the opposite side of the cell, TagA localizes to the distal end and prevents further polymerization, which would otherwise bend the sheath and cause its detachment from the membrane anchor [97, 98]. In ES401, overexpression of the VFFQA001_15530 homologue (TssA_VF_) inhibits T6SS activity [94], which is consistent with observations of TagA overexpression preventing sheath assembly in *V. cholerae* [98]. A Tn-insertion mutant of *tssA_VF_* fails to form sheaths and cannot inhibit growth of ES114 [85], which is surprising because *tagA* mutants of other bacterial species exhibit altered sheath assembly dynamics and retain some antibacterial activity. However, expression of the *tssA_VF_ in trans* failed to complement the killing activity unless expressed with downstream genes *tssM* and *tasR*, which suggests these other factors are likely affected by the transposon insertion. The C-terminal end of TssA_VF_ (and VFFQA001_15530) shows less than 15 % identity to other reported TagA homologues [85], which raises the possibility of a novel mechanism to control assembly dynamics of the inner tube/sheath complex. Smith *et al.* also reported that residues 285–288 (NKDD) correspond to a motif associated with GTP binding ([T/N]KxD) [85]. In GTPases, only the aspartate interacts with nucleotide; the asparagine and lysine residues stabilize interactions that the Walker A region has with the α- and β-phosphates [99]. Because TssA_VF_ does not feature a Walker A motif, additional biochemical studies of this factor are necessary to test whether it can bind GTP or other nucleotides.

### Impact of cellular aggregation on T6SS activity

For the T6SS to deliver effectors from one cell to another cell, both cells must be in direct contact. Consequently, factors that promote intercellular contact have been the subjects of recent studies. During the aggregation stage of initial light organ colonization, *V. fischeri* cells are exposed to a mucous matrix on the light organ surface, which is an environment predicted to exhibit high viscosity (Fig. 2) [68]. To determine how *V. fischeri* generally responds to viscous conditions, cells were exposed to medium that was supplemented with polymer polyvinylpyrrolidone (PVP) [84]. PVP is a water-soluble polymer composed of repeating *N*-vinylpyrrolidone monomers and has the property of increasing the viscosity of a solution to generate a so-called ‘hydrogel’. *V. fischeri* does not use PVP as a carbon source; however, upon exposure to PVP at 5 % (w/v) within liquid medium, *V. fischeri* cells form aggregates, with the aggregates becoming larger as the culture grows [84]. Examination of ES401 and other *V. fischeri* strains in this condition revealed that the ability to aggregate and kill ES114 in PVP depends on the presence of the T6SS2 gene cluster. For T6SS2-positive strains like ES401, exposure to PVP also results in transcriptional activation of *hcp*, sheath formation and the ability to kill ES114 [84]. A proteomics analysis of cellular protein did not detect increased abundance of T6SS-related factors after 12 h of PVP treatment [84], which is surprising as other results reported in this study clearly demonstrated T6SS activity by this time point. However, Hcp, VgrG and other T6SS core components were detected by 24 h [84].

Environmental pH also affects how certain strains aggregate following PVP treatment [85]. Relative to pH 7.5, an environmental pH of 8.2 lowers the extent of aggregation for ES401 but not for ES114, which in turn decreases the ability of ES401 to kill ES114 in co-culture likely by lowering the frequency of cell–cell contact [94]. This effect of pH on killing only occurred in the presence of PVP, as normal killing was observed when the strains were co-cultured on solid surface. This finding, i.e. a lower environmental pH is correlated with greater aggregation in PVP, is of potential relevance to symbiosis because *V. fischeri* is anticipated to transition from a pH of 8.2 in seawater to a pH of 6.4 within the mucus layer along the surface of the light organ ciliary appendages [68]. Planktonic cells are unable to increase T6SS activity at basic pH, even when exposed to calcium supplementation [100], which further highlights the importance of neutral or acidic pH for T6SS-dependent killing to occur. Notably, the aggregates that form outside of the light organ become enriched with *V. fischeri* over time [64]. The mechanisms underlying this winnowing remain unclear, but T6SS activity provides a mechanism for *V. fischeri* to winnow other cells prior to host entry. Experiments that move beyond PVP are warranted to test whether the effects occur in more biologically relevant conditions, e.g. in the mucins produced by *E. scolopes* that contain *N*-acetylneuraminic acid and *N*-acetylgalactosamine [67]. Furthermore, determining whether the pathways known to promote aggregation in *V. fischeri* are involved in the structures that form from PVP treatment are necessary to make ties with the cellular aggregates that form during initial steps of light organ colonization.

Proteomics analysis of PVP treatment has also revealed a novel factor associated with T6SS2. In FQ-A001, gene *VFFQA001_15520* is predicted to encode a large (~380 kDa) lipoprotein. Expression of its homologue in ES401, TasL, was detected after 24 h of PVP treatment [85]. Because TasL lacks a localization of lipoprotein avoidance signal, which canonically requires the presence of an aspartate at position 2 [101], the N terminus of TasL was proposed to be periplasmic, with the remaining portion of the protein extending into the extracellular environment [86, 102]. Consistent with this hypothesis, the C terminus features five repeat sequences that resemble domains found in the extracellular matrix proteins of the plasma fibronectin type III family [86, 103]. Based on *in silico* structural analysis, this lipoprotein is predicted to extend up to 70 nm from the cell surface [85]. A *tasL* mutant of ES401 could co-colonize light organ crypts with ES114, which suggests that TasL also promotes strain incompatibility [85]. Only a subset of *V. fischeri* strains are able to form co-aggregates with T6SS2-positive strain ES401 in media treated with PVP [86]. Disruption of *tasL* in ES401 abolishes co-aggregation, which suggests that TasL promotes cell contact between ES401 and certain *V. fischeri* strains [86]. These findings led to the hypothesis that TasL engages the competitor cell with some level of molecular specificity, but this remains to be tested [85].

Recently, calcium (Ca^2+^) has been shown to promote aggregation by *V. fischeri* [100, 104, 105]. The concentration of Ca^2+^ is approximately 10 mM in seawater [106], which indicates that *V. fischeri* routinely experiences this cation in its natural environment. A 15 h exposure of co-cultures of ES401 and ES114 to 10 mM Ca^2+^ but not Na^+^ or Mg^2+^ increases the recovery of ES401 relative to ES114, which suggests that calcium specifically alters the interaction between the two strains [104, 105]. Evaluation of the co-cultures by microscopy revealed that calcium increases cellular aggregation, and for T6SS2-positive strain ES401, also increases sheath assembly [100], which suggests a potential way to stimulate T6SS assembly. Future work is necessary to pursue this PVP-independent way to promote aggregation in *V. fischeri.*

## Regulatory mechanisms controlling T6SS expression

Like many bacteria, *V. fischeri* encodes the alternative sigma factor RpoN (σ^54^), which controls motility, bioluminescence and biofilm formation in *V. fischeri* [78, 107]. An in-frame *rpoN* mutant of FQ-A001 does not inhibit the growth of ES114 *in vitro* [78], which revealed that σ^54^ plays a role in controlling T6SS activity in *V. fischeri* as well. The two genes encoding Hcp (*hcp* and *hcp1*) in *V. fischeri* each feature canonical −24 (GG) and −12 (TGC) σ^54^-binding sites upstream of their respective coding sequences, and the promoters of each *hcp* gene exhibit low transcriptional activity in an *rpoN* mutant [78], which suggests that σ^54^ promotes transcriptional activation of the corresponding operons. Auxiliary gene cluster 3 does not feature any σ^54^-binding sites, which provides bioinformatic support that the corresponding genetic factors are not regulated by σ^54^. Within the main T6SS2 gene cluster, only the region upstream of the *hcp* gene features σ^54^-binding sites, which suggests σ^54^-dependent regulation is restricted to this operon and auxiliary gene cluster 2. In *V. cholerae *, there are two *hcp* genes that are regulated by σ^54^, and they are located within auxiliary gene clusters [108], which physically separates the genes associated with the inner tube and effectors away from the main T6SS gene cluster. Such an arrangement enables cells to differentially express most of the structural components from the factors that are secreted. The structural components of the T6SS other than Hcp and VgrG are frequently recycled [109], which enables the same subunits to contribute to multiple translocation events. In contrast, the secreted components must be synthesized *de novo*, and the genetic arrangement described above has been proposed as a way for cells to conserve energy by tightly regulating their expression [110]. In contrast to the main gene cluster in *V. cholerae *, that of T6SS2 in *V. fischeri* includes the σ^54^-regulated operon encoding Hcp and other putative effectors (Fig. 1b). Determining the impact of this genetic arrangement on regulation of T6SS2 will be important for understanding how *V. fischeri* expresses all the factors for a functional T6SS.

Because σ^54^ is necessary for FQ-A001 to colonize *E. scolopes* [78], it was not possible to use the *rpoN* mutant to directly assess the role of σ^54^ in regulating T6SS *in vivo*. As an alternative approach, the authors searched the T6SS2 gene cluster for a bacterial enhancer binding protein (bEBP), which is the type of transcription factor that is essential to initiate σ^54^-dependent transcription. In its active form, a bEBP overcomes the inhibition of open complex formation initially imposed by σ^54^ following recruitment of RNA polymerase (RNAP) to a σ^54^-dependent promoter [108, 111, 112]. Typically, bEBPs bind to enhancer sequences upstream of σ^54^-dependent promoters, form hexamers that facilitate ATPase activity, and interact directly with σ^54^-RNAP-promoter complexes to facilitate transcription. Most bEBPs feature an N-terminal regulatory domain, a central AAA^+^ (ATPases associated with various cellular activities) domain, and a C-terminal DNA-binding domain [111]. The *VFFQA001_15615* gene within the T6SS2 gene cluster encodes such a bEBP, with its regulatory domain (residues 25–180) comprising a GAF (cGMP-specific phosphodiesterases, adenylyl cyclases and FhlA) domain [113]. VFFQA001_15615 is referred to as VasH because its overall domain structure resembles that of VCA0117 (VasH), which is the bEBP that controls σ^54^-dependent transcription of the auxiliary T6SS genes in *V. cholerae* [114]. Recently, the GAF domain of VCA0117 was shown to interact with Hcp, so that transcription of *hcp* genes lowers if Hcp accumulates within the cytoplasm [110]. This negative feedback facilitated by VasH sensing intracellular Hcp ensures that the expression of secreted factors will cease if T6SS activity becomes impaired.

In *V. fischeri *, VasH is important for cells to exhibit T6SS activity, as culture-based assays revealed that a *vasH* mutant of FQ-A001 does not transcriptionally express either *hcp* gene and does not kill ES114 [78]. In contrast to the *rpoN* mutant, the *vasH* mutant colonizes the host to wild-type levels, which suggests that VasH does not regulate colonization factors in *V. fischeri *. Because the *vasH* mutant could colonize the host, the role of VasH in regulating Hcp expression could be assessed *in vivo*. By using GFP reporters for the promoter regions of both the *hcp* genes, researchers found that the mutant exhibits levels of *hcp* and *hcp1* expression lower than wild-type cells, which provided evidence that T6SS is expressed inside of the host [78]. The GAF domain of the VasH homologue in *V. fischeri* has yet to be examined for the ability to interact with Hcp. However, consistent with the VasH-dependent mechanism proposed to conserve energy in *V. cholerae* [110], transposon insertions within T6SS structural genes, e.g. *tssM* and *tssA_VF_*/*tagA*, result in mutants that display low levels of *hcp* expression when grown in hydrogel conditions [94].

The gene *VFFQA001_15540* encodes a 149-residue transcription factor called TasR (type VI-associated regulator) that is predicted to be a member of the Lrp/AsnC family of regulators. In *E. coli *, Lrp is a global regulator of genes involved in amino acid metabolism and transport [115]. The HTH_ASNC domain that comprises residues 9–115 of TasR consists of an N-terminal helix-turn-helix motif that interacts with DNA and a C-terminal domain that binds a small molecule ligand (e.g. leucine in Lrp of *E. coli *). Based on similarity to Lrp, TasR is predicted to form dimers that potentially oligomerize into higher-order structures that interact with DNA [116]. TasR is not encoded by *V. cholerae *, but in *Vibrio parahaemolyticus* it is encoded by *VP1407* within the T6SS1 gene cluster, which is associated with pathogenic isolates and exhibits a similar genetic arrangement to T6SS2 in *V. fischeri* [57, 117]. A *VP1407* mutant of *V. parahaemolyticus* fails to secrete Hcp and cannot kill other strains [118], which indicates that VP1407 is necessary for T6SS activity in the pathogen. In ES401, a transposon insertion resulted in a 19-residue C-terminal extension of TasR and eliminated both sheath formation and the ability to kill ES114 *in vitro* [94]. This *tasR* mutant also exhibits lower transcriptional activity of the *hcp* promoter [94], which suggests TasR is a positive regulator of T6SS2 in *V. fischeri *, like *VP1407* in *V. parahaemolyticus *. Expression of *tasR in trans* restored the ability of the *tasR* mutant to kill ES114 *in vitro* [94], which demonstrated genetic complementation. Determining how TasR regulates the *hcp* promoter and whether the corresponding mechanism involves VasH and σ^54^ will be important to gain insight into the regulation of T6SS in *V. fischeri *, and this knowledge will likely inform how TasR homologues functions in other bacteria like *V. parahaemolyticus *.

## Concluding remarks

The T6SS has been studied extensively in multiple bacterial species, and most of our current understanding comes from pathogens [14–26]. The recent discoveries highlighted in this review have increased understanding of how the T6SS impacts symbiosis establishment. Findings from multiple experimental studies have led to a model in which T6SS2-positive strains of *V. fischeri* can inhibit other strains from occupying the same habitat within the squid light organ (Fig. 3). This makes the T6SS an important factor to consider when studying the molecular mechanisms that impact the composition of symbiotic populations. However, there are still many unanswered questions. For example, the mechanism of killing by the T6SS depends on toxic effectors being translocated into the target cell [42]; however, to our knowledge, no cargo or specialized effectors have been functionally characterized in *V. fischeri *. While bioinformatics enables the detection of putative effector genes, such as those encoded downstream of a predicted DUF2143-containing chaperone protein (Fig. 1b), no effectors have been phenotypically evaluated, which presents a gap in knowledge about the mechanisms by which T6SS-sensative cells are eliminated. It also remains the case that, while a number of strains have been shown to harbour the complete T6SS gene cluster and inhibit ES114 growth in culture, only two strains (FQ-A001 and ES401) exhibit strain incompatible with ES114 *in vivo* [77, 79, 85]. Therefore, many opportunities remain to study the impact of the T6SS on competitor strain colonization dynamics. For example, studies that assess the population structure resulting from colonization of a host by two T6SS2-positive strains have the potential to reveal new symbiont dynamics *in vivo*. In summary, future research should focus on improving molecular and cellular insight into the T6SS of *V. fischeri* to further explore the important foundational observations highlighted in this review.
