# Erratum: The type-VI secretion system of the beneficial symbiont *Vibrio fischeri*

**DOI:** 10.1099/mic.0.001317

**Published:** 2023-03-03

**Authors:** Kirsten R. Guckes, Tim I. Miyashiro

**Affiliations:** ^1^​ Department of Biochemistry and Molecular Biology, Pennsylvania State University, PA, USA; ^2^​ Microbiome Center, Huck Institutes of the Life Sciences, Pennsylvania State University, PA, USA

## Full-Text

In the published version of the article, there was an omission in the affiliations list:

Author Kirsten R. Guckes and Author Tim I. Miyashiro should have both had two affiliations.

Previously, the author and affiliation list appeared as below:

Kirsten R. Guckes^1^, Tim I. Miyashiro^1^

Affiliations:

^1^The Microbiome Center, Huck Institutes of the Life Sciences, Pennsylvania State University, PA, USA

The correct author and affiliation list is as follows:

Kirsten R. Guckes^1,2^, Tim I. Miyashiro^1,2^

Affiliations:

^1^Department of Biochemistry and Molecular Biology, Pennsylvania State University, PA, USA

^2^Microbiome Center, Huck Institutes of the Life Sciences, Pennsylvania State University, PA, USA

The publishers apologise for this oversight.

